# Plasma Extracellular Vesicle Long RNAs Have Potential as Biomarkers in Early Detection of Colorectal Cancer

**DOI:** 10.3389/fonc.2022.829230

**Published:** 2022-04-08

**Authors:** Tian-An Guo, Hong-Yan Lai, Cong Li, Yan Li, Yu-Chen Li, Yu-Tong Jin, Zhao-Zhen Zhang, Hao-Bo Huang, Sheng-Lin Huang, Ye Xu

**Affiliations:** ^1^ Department of Colorectal Surgery, Fudan University Shanghai Cancer Center, Shanghai, China; ^2^ Shanghai Key Laboratory of Medical Epigenetics, the International Co-Laboratory of Medical Epigenetics and Metabolism, Ministry of Science and Technology, Institutes of Biomedical Sciences, Fudan University, Shanghai, China; ^3^ Department of Oncology, Shanghai Medical College, Fudan University, Shanghai, China; ^4^ Department of Biostatistics and Bioinformatics, Emory University, Atlanta, GA, United States; ^5^ Department of Endoscopy, Fudan University Shanghai Cancer Center, Shanghai, China; ^6^ Department of Blood Transfusion, Fujian Medical University Union Hospital, Fuzhou, China

**Keywords:** extracellular vesicle, long RNAs, colorectal cancer, colorectal adenoma, early detection

## Abstract

**Background:**

Early detection of colorectal cancer (CRC) is crucial to the treatment and prognosis of patients. Traditional screening methods have disadvantages.

**Methods:**

231 blood samples were collected from 86 CRC, 56 colorectal adenoma (CRA), and 89 healthy individuals, from which extracellular vesicle long RNAs (exLRs) were isolated and sequenced. An CRC diagnostic signature (d-signature) was established, and prognosis-associated cell components were evaluated.

**Results:**

The exLR d-signature for CRC was established based on 17 of the differentially expressed exLRs. The d-signature showed high diagnostic efficiency of CRC and control (CRA and healthy) samples with an area under the curve (AUC) of 0.938 in the training cohort, 0.943 in the validation cohort, and 0.947 in an independent cohort. The d-signature could effectively differentiate early-stage (stage I–II) CRC from healthy individuals (AUC 0.990), as well as differentiating CEA-negative CRC from healthy individuals (AUC 0.988). A CRA d-signature was also generated and could differentiate CRA from healthy individuals both in the training (AUC 0.993) and validation (AUC 0.978) cohorts. The enrichment of class-switched memory B-cells, B-cells, naive B-cells, and mast cells showed increasing trends between CRC, CRA, and healthy cohorts. Class-switched memory B-cells, mast cells, and basophils were positively associated with CRC prognosis while natural killer T-cells, naive B-cells, immature dendritic cells, and lymphatic endothelial cells were negatively associated with prognosis.

**Conclusions:**

Our study identified that the exLR d-signature could differentiate CRC from CRA and healthy individuals with high efficiency and exLR profiling also has potential in CRA screening and CRC prognosis prediction.

## Introduction

Colorectal cancer (CRC) ranks the third common cancer in men and the second in women, as well as the second cause of cancer death worldwide, which remains an enormous socioeconomic burden on society (1, 2). Meanwhile, colorectal adenoma (CRA) usually take years to develop to invasive or metastatic CRC, which makes CRC one of the cancers most suitable for early detection (3).

Early detection of CRC is the key to reducing invasive treatment, morbidity, mortality, and treatment cost (3). CRC screening methods include invasive and non-invasive tests. Colonoscopy is widely known as the golden standard but limited by invasiveness and low compliance rate (4). The guaiac fecal occult blood test (gFOBT) and fecal immunochemical test for hemoglobin (FIT) are most widely used because they are convenient, cheap, and non-invasive. However, these fecal tests have limitations of low sensitivity or specificity (3). CT colonography, anther non-invasive test, is costly and not sensitive to tumors less than 10 mm (3, 5). From the above, blood tests tend to be more acceptable for CRC screening, but no reliable detecting method or markers have been widely acknowledged (6).

Extracellular vesicles (EVs) are extracellular membrane vesicles originated and released from endocytosis and exocytosis, containing proteins, DNA, RNA, and lipids (7). Due to the protection of the lipid membrane, EV RNAs are likely to be more stable than other free plasma RNA. Long RNAs have been identified in human blood EVs, including messenger RNA (mRNA), long non-coding RNA (lncRNA), and circular RNA (circRNA), which have emerged as promising markers for cancer diagnosis recently and have already been evaluated in some cancers (8–10). However, difficulties in EV research lie on the lack of efficient and stable methods for plasma EVs isolating and purifying. Fortunately, an optimized strategy for plasma EV long RNA (exLR) sequencing (exLR-seq) has been developed and reliable positive data have been obtained in our recent studies (11, 12).

In this study, a CRC diagnostic signature (d-signature) based on plasma exLR profiling was identified and validated, which could differentiate CRC from control (CRA and healthy) individuals efficiently. We also evaluated cell components and signaling pathways between CRC, CRA, and healthy groups, and associated prognostic significance were revealed.

## Patients and Methods

### Patients

From February 2018 to January 2019, 194 blood samples were collected from 72 CRC patients, 42 CRA patients, and 80 age- and sex-matched healthy participants receiving routine medical examination. The diagnoses of all CRC and CRA patients were pathologically confirmed, and these participants did not have a history of other malignant tumors. All enrolled CRC patients underwent surgical treatment without preoperative chemotherapy or radiotherapy at the Colorectal Surgery Department of Fudan University Shanghai Cancer Center. 37 blood samples (14 CRC, 14 CRA, 9 healthy) were collected in an independent center from Fujian Medical University Union Hospital.

### EVs Identification and exLR-seq Analysis

The optimized strategy for plasma exLR-seq included several steps as follows: plasma sample collection, EV purification, transmission electron microscopy (TEM), size distribution measurement, RNA isolation, and RNA-seq library preparation (11). To be brief, the blood samples of CRC and CRA patients were collected before the excision of tumor and centrifuged twice at 3,000 and 13,000 rpm, respectively. The EV RNAs were isolated using the exoRNeasy Serum/Plasma Kit, and the EVs were photographed using a TEM. The size distribution was analyzed using Flow NanoAnalyzer. EV markers TSG101 and CD63 were estimated by Western blots. The RNA-seq library was prepared using SMART technology and sequenced by the Illumina sequencing platform. Details of these steps are found in **Supplementary Materials.**

### ExLR-Seq Analysis for Quantifying Gene Expression

The qualified FASTQ files generated from RNA-seq were aligned to the human genome (hg38) using STAR v2.5.3 with default parameters (13). The mapped sequencing reads in the resulting BAM files were then assigned to genes by featureCounts v1.6.3 (14). Considering that the transcriptome library was reversely stranded, “-s” was set as 2 for performing strand-specific read counting. Genes were annotated with GENCODE v.29. The read count of each gene was converted to transcripts per million (TPM) as follows:


$$TPM_{i}=\frac{RC_{i}}{L_{i}}*\left(\frac{1}{\sum_{j=1}^{LR}\frac{RC_{j}}{L_{j}}}\right)*10^{6}$$

Where *RC_i_* stands for the count of reads mapped to the gene and *L_i_* is the length of the gene. *LR* is the number of long RNA genes including protein coding and long non-coding genes.

### Differential Expression Analysis and Pathway Enrichment Analysis

We calculated the correlation coefficient between each two samples based on TPM expression profiles and filtered poor samples with the median of correlation coefficients smaller than 0.9. The final dataset analyzed in our study contained 72 CRC samples and 122 control (42 CRA and 80 healthy) samples. To explore differentially expressed genes (DEGs) between these two cohorts, we applied R package “limma” on TPM expression profiles (15). The Benjamini–Hochberg approach was used to adjust the *p* values for multiple testing. A gene with a fold change (FC) bigger than 1.5 and adjusted *p* value smaller than 0.05 was defined as a DEG. To investigate the differential pathways between CRC and control samples, R package “clusterProfiler” was used for KEGG pathway enrichment analysis based on the DEGs (16).

### Selecting Effective Feature Genes and Building CRC/CRA-Identification Model

The whole dataset was randomly divided into training cohort (48 CRC and 82 control) and validation cohort (24 CRC and 40 control). With respect to the training cohort, we firstly conducted DEG analysis. To elect informative and functional signature genes for effectively distinguishing CRC samples from control samples, we focused on these upregulated protein coding or long non-coding genes in CRC samples. Then, we employed the minimum redundancy maximum relevance (mRMR) algorithm to rank these candidate genes. This was implemented using the mRMR package with the “MIQ” feature selection scheme (http://home.penglab.com/proj/mRMR/) (17). Next, we applied the incremental feature selection (IFS) strategy to determine the optimal subset of feature genes based on the support vector machine (SVM) (18). The first feature set was constructed with the top one gene. The remaining ranked feature genes were added one by one incrementally for producing new feature sets. Each new feature set was composed of the previous set adding with a new feature gene. Each feature gene set was evaluated with the area under the curve (AUC) value derived from the SVM model using leave one out cross-validation (LOOCV). Finally, the optimal CRC-identification model was built using the feature gene set with the highest AUC value. This model was then applied to classify the validation cohort for further assessing the prediction performance of these feature genes. SVM models were constructed using the LibSVM software package downloaded from https://www.csie.ntu.edu.tw/~cjlin/libsvm/ (19). The CRA-identification model was built in the same way.

### Cell Type and Pathway Estimation

To infer the cell types of EV origins, we performed xCell analysis on TPM expression profiles using R package “xCell,” a gene signature-based method that integrates the advantages of gene set enrichment with deconvolution approaches (20). We obtained the enrichment scores of 64 immune and stromal cell types and further investigated the influence of each cell type on the overall survival (OS) and disease-free survival (DFS) of CRC samples. The survival analysis and Kaplan–Meier plotting were implemented by R package “survminer.” The single sample gene set enrichment analysis (ssGSEA) algorithm was used to calculate the enrichment scores of the canonical MSigDB pathways (C2, KEGG) (21). This was carried out on R package “GSVA” with the method of “ssGSEA” (22). To explore the significant different cell types and pathways among CRC, CRA, and normal cohorts, the Wilcoxon-rank sum test was used for comparison between any two cohorts and the one-way analysis of variance (ANOVA) test was used for comparisons among the three cohorts.

## Results

### Patient Characteristics

In general, 194 participants were involved in our center, consisting of 72 CRC patients, 42 CRA patients, and 80 healthy individuals. The clinicopathological information is listed in **Table 1**. No obvious difference was seen in age, gender, or tumor site between the three groups. We included more early-stage CRC (stage I–II, 53 cases) than advanced CRC (stage III–IV, 19 cases) because this study was designed to mainly focus on the early detection of CRC. All the CRC patients were followed up for at least 24 months. Death events were observed in 13 stage IV CRC patients, and tumor recurrence or metastasis events were observed in 8 stage II/III CRC patients.

**Table 1:** Clinicopathological information of 194 participants.

	CRC (N = 72)	CRA (N = 42)	Healthy (N = 80)
Age	60.8 ± 10.9	56.2 ± 10.7	59.9 ± 13.0
Gender			
Male	48	24	54
Female	24	18	26
Tumor site			
Right colon	15	11	NA
Left colon	23	13	NA
Rectum	34	18	NA
TNM stage			
I	22	NA	NA
II	31	NA	NA
III	3	NA	NA
IV	16	NA	NA

NA, not available.

### EVs Isolation and exLR-seq

**Figure 1:**
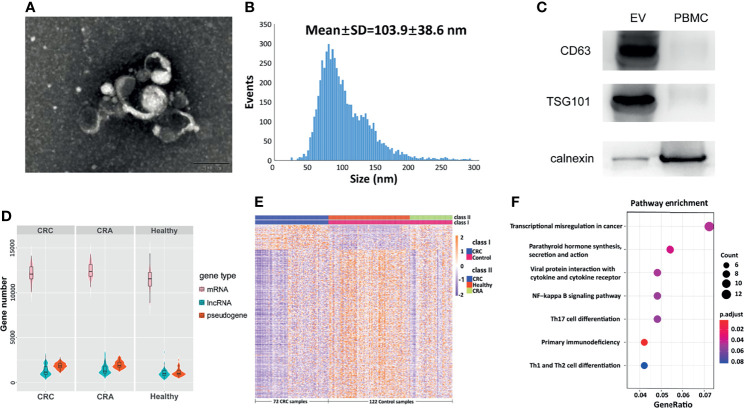
Plasma EVs and exLR-seq. **(A)** Photograph of EVs using a TEM. **(B)** Size distribution of EVs. **(C)** Western blot analysis of EV markers TSG101 and CD63 in PMBC and EVs. **(D)** Amount of exLRs for each sample among CRC, CRA, and healthy individuals. **(E)** Unsupervised hierarchical clustering of the exLRs differentially expressed between CRC and control (class I); CRC, healthy, and CRA (class II). **(F)** KEGG pathway enrichment analysis for differentially expressed exLRs.

The isolated EVs observed by TEM were round capsule bubbles. The scanning electron microscope images of EVs are shown in **Figure 1A**. Since types of EVs (exosomes, microvesicles, and apoptotic bodies) should be distinguished by diameter, we analyzed the size distribution by flow cytometry (10). The size distribution result revealed abundant peaks ranging from 50 to 200 nm and a mean diameter of 103.9 ± 38.6 nm (**Figure 1B**), indicating that morphologically most of the isolated EVs were exosomes with definition of 40–200 nm in diameter (10). Western blot analysis confirmed that the EV markers CD63 and TSG101 were enriched in EVs but not peripheral blood mononuclear cells (PMBCs), while the negative-control protein marker calnexin was enriched in PMBCs but not EVs (**Figure 1C**). Afterward, exLR-seq was conducted and no obvious difference of detected mRNA, lncRNA, and pseudogene amount was observed between the three groups (**Figure 1D**). Unsupervised hierarchical clustering revealed clear separations of CRC and control (CRA and healthy) samples, as well as CRC, CRA, and healthy samples (**Figure 1E**). The differentially expressed exLRs were enriched for some cancer-associated pathways, such as transcriptional misregulation in cancer and NF-kappa B signaling pathway (**Figure 1F**). Therefore, we hypothesized that exLRs have potential as diagnostic biomarkers of CRC.

### Establishment of an exLR d-Signature for CRC

**Figure 2:**
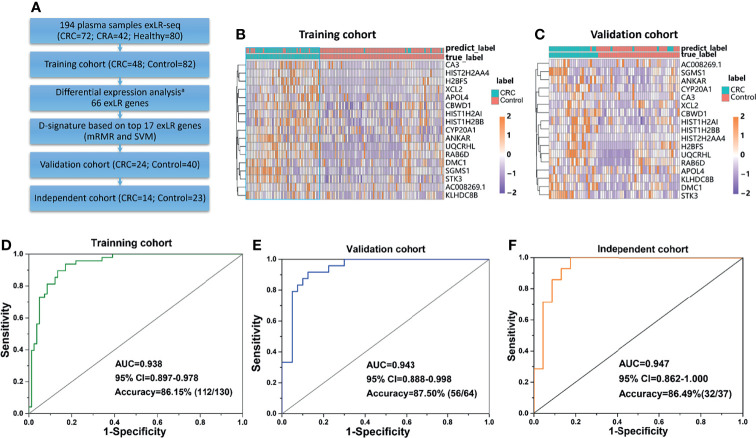
Establishment of the exLR d-signature. **(A)** Flowchart of establishment of the d-signature. **(B, C)** Unsupervised hierarchical clustering of the 17 genes in training cohort **(B)** and validation cohort **(C)**. **(D–F)** ROC curve for the exLR d-signature in the training **(D)**, validation **(E)**, and independent **(F)** cohorts. ^a^Selection of lncRNA or protein-coding genes with (1) expression frequency >0.5; (2) log2(FC) >0.59, adjusted p value < 0.05.

To identify the diagnostic potential of exLRs, we developed an exLR-based d-signature for CRC. The flowchart of the establishment of the d-signature is presented in **Figure 2A**. By random sampling, the cohort was divided into a training cohort (48 CRC, 82 control) and a validation cohort (24 CRC, 40 control). Next, we selected 66 long RNA genes upregulated in CRC samples compared with control samples by DEG analysis (expression frequency >0.5, log_2_(FC) >0.59 and adjusted *p* value < 0.05). MRMR and SVM were used to select the optimal feature gene set among the training cohort. The top 17 genes of the ranked 66 genes were selected to build the SVM prediction model (**Table 2**). Unsupervised hierarchical clustering of the 17 genes showed relatively high consistency between predicting CRC and true CRC individuals in both training and validation cohorts (**Figures 2B, C**). The d-signature was applied in the training cohort and validation cohort to assess the diagnostic efficiency. We generated receiver operating characteristic (ROC) plots, displaying the performance of the d-signature in the training cohort, the validation cohort, and the independent cohort (**Figures 2D–F**). The training sensitivity, specificity, and accuracy were 82.93%, 93.75%, and 86.15%, respectively (**Figure 2D** and **Table 3**). The validation sensitivity, specificity, and accuracy were 87.50%, 91.67%, and 87.50%, respectively (**Figure 2E** and **Table 3**). The sensitivity, specificity, and accuracy of the independent cohort were 71.43%, 95.65%, and 86.49% (**Figure 2F** and **Table 3**). The CRC d-signature showed high diagnostic efficiency both in the training cohort and the validation cohort, as well as the independent cohort.

**Table 2:** Basic information and expression of the 17 feature genes.

Gene ID	Gene name	Gene type	Expression frequency	log2(FC)	Mean CRC	Mean control
ENSG00000272196.2	HIST2H2AA4	Protein coding	0.77	1.92	21.79	5.17
ENSG00000234289.5	H2BFS	Protein coding	0.67	1.65	13.91	5.45
ENSG00000233954.6	UQCRHL	Protein coding	0.97	1.49	17.41	8.23
ENSG00000143185.3	XCL2	Protein coding	0.73	1.32	32.43	18.73
ENSG00000229321.1	AC008269.1	lncRNA	0.53	1.01	15.96	9.4
ENSG00000100206.9	DMC1	Protein coding	0.86	0.98	17.2	9.75
ENSG00000233087.7	RAB6D	Protein coding	0.90	0.87	3.68	1.82
ENSG00000185909.14	KLHDC8B	Protein coding	0.82	0.84	15.09	9.76
ENSG00000164879.6	CA3	Protein coding	0.63	0.82	7.54	4.44
ENSG00000100336.17	APOL4	Protein coding	0.81	0.76	7.69	4.45
ENSG00000196747.4	HIST1H2AI	Protein coding	0.97	0.76	235.37	165.53
ENSG00000151687.14	ANKAR	Protein coding	0.95	0.74	16.07	9.88
ENSG00000198964.13	SGMS1	Protein coding	0.99	0.71	76.67	45.6
ENSG00000119004.15	CYP20A1	Protein coding	0.93	0.68	19.07	13.9
ENSG00000276410.3	HIST1H2BB	Protein coding	0.99	0.66	497.04	293.11
ENSG00000104375.16	STK3	Protein coding	0.99	0.63	19.03	12.62
ENSG00000274559.3	CBWD1	Protein coding	0.97	0.61	10.65	7.42

FC, fold change.

**Table 3:** The exLR d-signature in diagnosis of CRC, CRA, and healthy participants.

Groups	AUC	95% CI	Sensitivity (%)	Specificity (%)	Accuracy (%)
Training	0.938	0.897–0.978	77.08	91.46	86.15
Validation	0.943	0.867–0.995	91.67	85.00	87.50
Independent cohort	0.947	0.801–1.000	71.43	95.65	86.49
CRC vs. CRA	0.853	0.776–0.930	76.19	84.72	79.83
CRC vs. healthy	0.983	0.969–0.997	92.50	94.44	89.47
Stage I/II CRC vs. CRA	0.882	0.809–0.955	85.71	81.13	82.11
Stage I/II CRC vs. healthy	0.990	0.979–1.000	95.00	96.23	92.48
CEA-negative CRC vs. CRA	0.870	0.790–0.950	76.19	87.81	80.72
CEA-negative CRC vs. healthy	0.988	0.974–1.000	92.50	97.56	92.56
CRA vs. healthy training	0.993	0.981–1.000	89.29	98.15	95.12
CRA vs. healthy validation	0.978	0.940–1.000	71.43	96.15	87.50

AUC, area under the curve; CI, confidence interval; CEA, carcinoembryonic antigen.

### The exLR d-Signature for Early Detection of CRC

**Figure 3:**
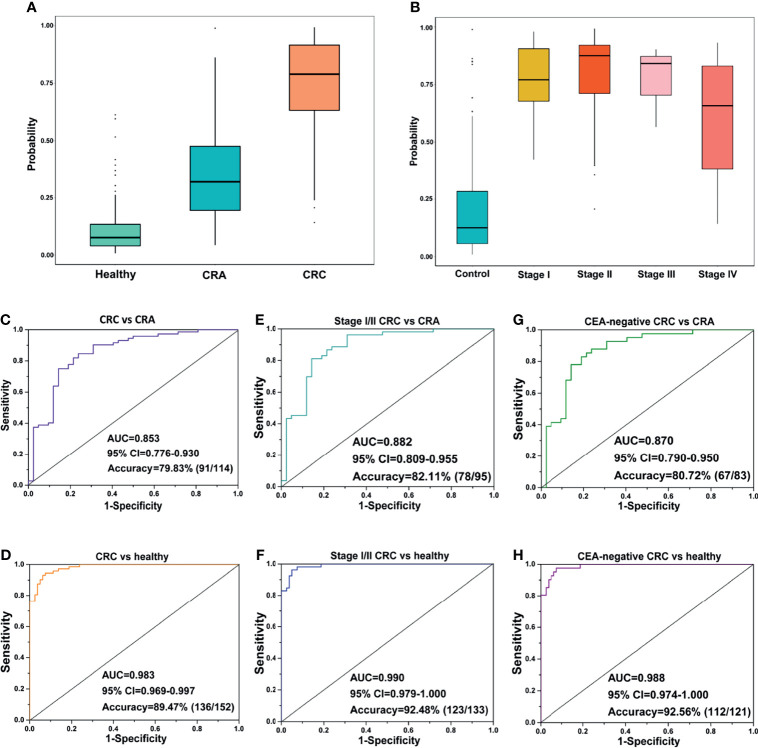
Prediction performance of the exLR d-signature in subgroups. **(A)** The d-signature in distinguishing healthy, CRA, and CRC individuals. **(B)** The d-signature in control and stage I–IV CRC participants. The ROC curve for the d-signature in CRC and CRA **(C)**, CRC and healthy **(D)**, early-stage (stage I–II) CRC and CRA **(E)**, early-stage (stage I–II) CRC and healthy **(F)**, CEA-negative CRC and CRA **(G)**, and CEA-negative CRC and healthy **(H)** cohorts.

We further evaluated the performance of the exLR d-signature in subgroups. The d-signature could differentiate between healthy, CRA, and CRC cohorts, and an increasing trend of the diagnostic probability was shown among the three cohorts, which is consistent with the process of CRC carcinogenesis (**Figure 3A**). Performance of the d-signature was then assessed among different stages of the CRC and control cohorts. As shown in **Figure 3B**, the d-signature had diagnostic ability for CRC of stages I, II, III, and IV. The sensitivity, specificity, and accuracy of the d-signature to differentiate CRC from CRA were 76.19%, 84.72%, and 79.83% (**Figure 3C** and **Table 3**). The diagnostic efficiency was higher for the d-signature to differentiate between CRC and healthy cohorts (sensitivity 92.50%, specificity 94.44%, accuracy 89.47%, **Figure 3D** and **Table 3**). As for the early-stage (stage I–II) CRC versus CRA subgroup, the sensitivity, specificity, and accuracy were 85.71%, 81.13%, and 82.11% (**Figure 3E** and **Table 3**). The sensitivity, specificity, and accuracy for the d-signature to differentiate between early-stage (stage I–II) CRC and healthy cohorts were 95.00%, 96.23%, and 92.48%, respectively (**Figure 3F** and **Table 3**).

Carcinoembryonic antigen (CEA) is one of the most common cancer markers but limited by low diagnostic efficiency when used along for CRC diagnosis (23). The performance of the d-signature in distinguishing CEA-negative CRC from CRA cohorts is presented in **Figure 3G** and **Table 3** (sensitivity 76.19%, specificity 87.81%, accuracy 80.72%). High performance was observed of the d-signature to differentiate CEA-negative CRC from healthy cohorts (sensitivity 92.50%, specificity 97.56%, accuracy 92.56%, **Figure 3H** and **Table 3**). The diagnostic ability of the d-signature to differentiate between CRA and CRC, especially early-stage (stage I–II) and CEA-negative CRC, was of great significance to determine whether the tumor should be resected endoscopically or surgically in clinical practice. Meanwhile, the high efficiency of the d-signature to differentiate between healthy and CRC individuals, including early-stage and CEA-negative CRC individuals, was supposed to have an important potential role in CRC screening.

### Potential of the exLR d-Signature in Detecting CRA

**Figure 4:**
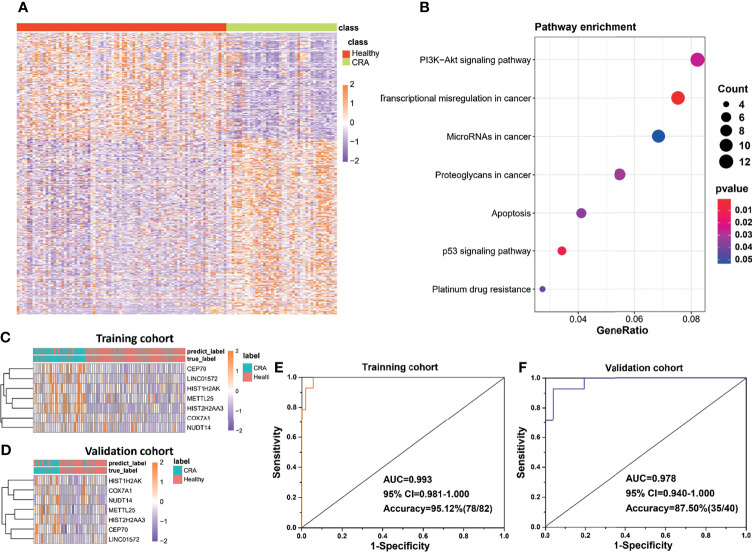
Potential of the exLR d-signature in differentiating CRA and healthy participants. **(A)** Unsupervised hierarchical clustering of the differentially expressed exLRs between CRA and healthy cohorts. **(B)** KEGG pathway enrichment analysis for the differentially expressed exLRs between CRA and healthy cohorts. **(C, D)** Unsupervised hierarchical clustering of the 7 genes selected for d-signature establishment in the training cohort **(C)** and validation cohort **(D)**. **(E, F)** ROC curve for the exLR d-signature in the training **(D)** and validation **(E)** cohorts.

In addition to the diagnosis of CRC, detection of CRA is also a very important link in the management of CRC, considering CRA as precancerous lesions of CRC. In this part, we developed another exLR-based d-signature for CRA in the same way as building the CRC d-signature. Unsupervised hierarchical clustering revealed a clear separation of CRA and healthy samples (**Figure 4A**). KEGG analysis showed that the differentially expressed exLRs were enriched for some tumor-associated pathways (**Figure 4B**). Unsupervised hierarchical clustering of the top 7 genes selected to build the CRA-identification model showed high consistency between predicting CRA and true CRA individuals in both the training and validation cohorts (**Figures 4C, D**). Encouraging results of the CRA d-signature were observed both in the training (sensitivity 89.29%, specificity 98.15%, accuracy 95.12%) and validation (sensitivity 71.43%, specificity 96.15%, accuracy 87.50%) cohorts (**Figures 4E, F** and **Table 3**).

### Estimation of Cell Populations and Prognostic Prediction

**Figure 5:**
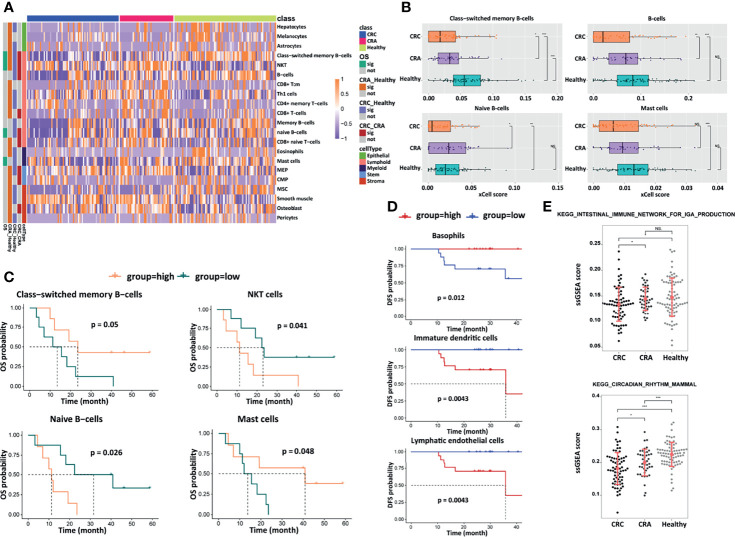
Analyses of cell components, survival, and signaling pathways. **(A)** Heatmap of unsupervised hierarchical clustering of the 21 cell types in different groups. **(B)** Box plots of selected cell-type abundance between CRC, CRA, and healthy groups. Prognostic significance of selected cell types by **(C)** OS and **(D)** DFS. **(E)** ssGSEA score and statistical significance for selected KEGG pathways differing between CRC, CRA, and healthy groups. ***p < 0.001; **p < 0.01; *p < 0.05; NS, not significant.

EVs are produced by many cell types including immune cells, serving as communicators of immune-modulatory activities that affect the tumor microenvironment and antitumor immune responses (24). We used xCell to infer cell populations represented in EVs. Abundances of 64 immune and stromal cell types based on gene expression profile were estimated, and 21 of them showed statistical differences, including epithelial, lymphoid, myeloid, stem, and stroma cells (**Figure 5A**). Low enrichment of class-switched memory B-cells, B-cells, naive B-cells, and mast cells was observed in the CRC group compared with CRA and healthy groups, and there was a slight increasing trend among CRC, CRA, and healthy cohorts, implying that the tumor-immune microenvironment had been affected in the CRC group (**Figure 5B**). In the analysis of prognostic significance, a positive correlation was observed between longer OS and the abundance of class-switched memory B-cells and mast cells, while a negative correlation was observed between OS and the abundance of natural killer T-cells (NKT cells) and naive B-cells (**Figure 5C**). A high basophil level was associated with longer DFS, while a high level of immature dendritic cells and lymphatic endothelial cells predicted shorter DFS (**Figure 5D**). These prognosis-associated cell populations were supposed to play a role in CRC prognostic prediction. Besides, we assessed the pathway enrichment of differentially expressed transcriptomes between the CRC, CRA, and healthy groups by performing gene set enrichment *via* KEGG analysis, showing that the differentially expressed exLRs were enriched in the intestinal immune network for the IgA production pathway and the circadian rhythm mammal pathway with a gradual rising trend between the three groups (**Figure 5E**). These results presented the potential applications of the exLR profiling.

## Discussion

In this study, exLR-seq expression profiles were gained from 231 CRC, CRA, and healthy blood samples. To our knowledge, this is the first study focusing on the early detection potential of exLRs between CRC, CRA, and healthy individuals. The preliminary findings seem to be inspiring as certain diagnostic and prognosis prediction efficiency was achieved.

Extracellular vesicles, known as small membranous vesicles released by cells, have recently been identified to contain long RNAs, which may serve as biomarkers in the diagnosis, therapeutic sensitivity prediction, and prognostic prediction of tumors (8, 9, 12, 25). Although the clinical application of EVs is still in its infancy, EVs are increasingly recognized as promising biomarkers for tumor diagnosis and prognosis (10). However, previous studies are mainly focused on protein and miRNAs in EVs. In reviewing the literature, no published study was found to in-depth analyze the diagnostic or prognostic value of exLRs in CRC due to the limitation of methodology and size of samples.

Nowadays, the incidence and mortality of colorectal cancer remain high in both developed and developing countries. Early detection is a key to reducing morbidity and the socioeconomic burden. Traditional detection methods, including colonoscopy, gFOBT, FIT, and CT colonography, all have some limitations of invasiveness, high expense, or low efficiency (2, 3). Emerging screening strategies, such as ctDNA, circulating tumor cells, and septin-9, have been studied widely. Nonetheless, results in relevant studies have shown much lower diagnostic efficiency of CRA and early-stage CRC than that of advanced-stage CRC (6, 26).

A diagnostic signature based on plasma exLR profiling was developed in this study. We first verified EVs from TEM morphology, size distribution analysis, and Western blot analysis. These all corresponded to the characteristics of EVs (7). ExLR profiling of plasma samples from 194 participants was successfully performed using an optimized exLR-seq strategy we recently developed (11). We established a d-signature of 17 exLRs for CRC detection, which could efficiently differentiate CRC from control (CRA and healthy) cohorts (training AUC = 0.938, validation AUC = 0.943, independent cohort AUC = 0.947). In clinical practice, people with positive testing results are supposed to take colonoscopy examination to identify the results. The d-signature makes it possible to screen high-risk patients efficiently and reliably, standing a good chance of easing the suffering of the screened people and improving screening compliance.

High sensitivity and specificity were identified for the d-signature to differentiate CRC from CRA, which was of great significance in clinical practice, especially when it comes to early-stage (stage I–II) CRC or CEA-negative CRC. In clinical practice, CRA patients need no additional surgery if the polyp has been completely endoscopically resected with favorable histologic features, while radical surgery plays a vital role in the treatment of most early-stage CRC patients (27, 28). Different diagnoses of CRC or CRA lead to different treatment strategies, and this d-signature is supposed to provide reference for clinicians and patients to make decisions. Compared with differentiating between CRA and CRC cohorts, the d-signature had higher diagnostic efficiency to differentiate between healthy and CRC cohorts, including early-stage (stage I–II) CRC and CEA-negative CRC. This is of great significance for improving the efficiency of CRC screening, considering the limitations of traditional non-invasive CRC screening methods (3, 5).

The 17 genes used to establish the d-signature comprised 16 protein-coding genes and one lncRNA gene, all of which were upregulated in CRC samples. The H2BFS expression level in lung cancer tissue has been reported to be higher than that in normal lung tissue (29). However, its expression in CRC remains unknown. In a previous study, a high expression level of XCL2 was revealed to be associated with NK cells in tumor-immune activities (30). DMC1, short for “downregulated in multiple cancers-1,” plays an important role in DNA binding and repairing, with loss expression identified in multiple human cancers (31). The different expression levels in this study might be explained by using peripheral blood samples but not tumor tissue samples. KLHDC8B is suggested to have a role in the formation of Hodgkin/Reed–Sternberg cells in familial Hodgkin lymphoma (32). CA3 expression is reported to promote the transformation and invasive ability of hepatocellular carcinoma cells (33). Overexpressed CYP20A1 is observed in some pathological types of lung cancer and associated with prognosis according to a previous study (34). The expression of HIST1H2BB is reduced in ovarian cancer cells and might have growth-suppressing roles (35). STK3 is a critical molecule of the Hippo pathway that controls cell development, proliferation, and apoptosis (36). The expression level of CBWD1 has been reported to be associated with melanoma (37). The tumor-associated significance of the other seven genes (HIST2H2AA4, UQCRHL, AC008269.1, RAB6D, APOL4, HIST1H2AI, ANKAR, SGMS1) remains unclear.

This study was mainly designed to build a d-signature for CRC screening, and we were surprised to find that a similar model might be very efficient in CRA diagnosis. However, due to the limitation of CRA cohort size, we believe that the encouraging initial results need to be reconfirmed in further study with larger cohorts.

In this study, statistical differences of 21 immune cell types estimated based on the gene expression profile were observed between CRC, CRA, and healthy cohorts. Actually, the relationship between systemic immune cells and CRC still remains poorly understood, even though some studies with a small sample size have yielded some preliminary conclusions (38, 39). In this study, differences in immune cell subset distribution were observed between CRC, CRA, and healthy cohorts, such as reduced percentage of class-switched memory B-cells, B-cells, naive B-cells, and mast cells in the CRC cohort. This study also showed correlations between survival and these cells. A decreased percentage of peripheral blood B-cells and naive-B cells in the CRC cohort compared with the healthy cohort has been reported previously, whereas the percentage of peripheral blood memory B cells was increased in the CRC cohort in that study (39). Contrary prognostic implications of class-switched memory B-cells and naive B-cells were revealed in this study, and both the tumor progression-enhancing and -suppressing effects of B-cells have been reported in previous literature (40, 41). Activation or suppression of B cells may play an important role in CRC carcinogenesis, which needs to be identified in further studies. The difference of peripheral blood mast cell count between CRC and healthy cohorts has not been reported, and its relationship with survival remains controversial (42, 43). High levels of NKT cells were related to poor prognosis in this study; a similar result has been reported previously (38). In a recent study, a decreased level of circulating basophils was found linked to aggressive biology and poor survival, which is similar to the result of this study (44). In this study, a high level of immature dendritic cells predicted poor survival. Actually, a dendritic cell-infiltrating level has been reported to be positively correlated with layilin and a high layilin level was linked to poor survival in colorectal cancer patients (45). A lymphatic endothelial cell level was associated with poor survival in this study. Lymphatic vessel invasion has been identified as an independent prognostic factor for poor survival in colorectal cancer, and CRC-associated intestinal lymphatic endothelial cells were revealed to be able to regulate tumor progression (46). Further studies are needed to evaluate the role of peripheral blood immune cells in CRC progression and the potential of EVs estimating peripheral blood immune cells.

Furthermore, differentially expressed exLRs between CRC, CRA, and healthy cohorts were enriched in two pathways, the intestinal immune network for the IgA production pathway and the pathway of circadian rhythm of mammal. IgA deficiency is associated with a number of immune-mediated diseases, and it has also been proved to be associated with increased risk of gastrointestinal cancer in a nationwide population-based cohort study (47). Circadian rhythms of cell cycle–related molecule expression have been extensively reported (48). In a recently published study, circadian disruption was revealed to be associated with tumor-associated immune cell remodeling, resulting in facilitation of tumor growth (49).

Limitations and prospects of this study are listed as follows. First, the independent cohort size was limited and the diagnostic performance of the CRC d-signature needs to be validated in more independent centers. Second, we are continuing to recruit participants to identify the efficiency of the CRA d-signature. Third, the potential of EVs in predicting chemotherapy resistance is under study.

In summary, our study evaluated the value of exLRs serving as markers in the detection of CRC. The d-signature we have established can differentiate CRC from control (CRA and healthy) cohorts efficiently, which is supposed to improve CRC early detection efficiency in clinical practice. The exLR profiling can also indicate immune cell distribution and associated prognostic significance. We believe that this d-signature can contribute to the early detection of CRC and improve CRC prognosis in the near future.

## Data Availability Statement

The datasets presented in this study can be found in online repositories. The names of the repository/repositories and accession number(s) can be found in the article/**Supplementary Material**.

## Ethics Statement

The studies involving human participants were reviewed and approved by the Ethics Committee of the Fudan University Shanghai Cancer Center. The patients/participants provided their written informed consent to participate in this study.

## Author Contributions

T-AG, H-YL, Z-ZZ, H-BH, S-LH, and YX were responsible for the study concept and study design. T-AG, H-YL, and CL performed the data acquisition. H-YL, Y-TJ, YL, and Y-CL were responsible for the methodology, software, formal analysis, and visualization. T-AG and H-YL wrote the original draft. YX, S-LH, and Z-ZZ edited and revised the manuscript. All authors contributed to the article and approved the submitted version.

## Funding

This work was supported by the National Natural Science Foundation of China (82072694, 81872294), the Shanghai Science and Technology Innovation Action Plan (20JC1419000), and the Shanghai Committee of Science and Technology (20DZ1100101, 19511121202).

## Conflict of Interest

The authors declare that the research was conducted in the absence of any commercial or financial relationships that could be construed as a potential conflict of interest.

## Publisher’s Note

All claims expressed in this article are solely those of the authors and do not necessarily represent those of their affiliated organizations, or those of the publisher, the editors and the reviewers. Any product that may be evaluated in this article, or claim that may be made by its manufacturer, is not guaranteed or endorsed by the publisher.

## Abbreviations

CRC, colorectal cancer; exLR, extracellular vesicle long RNA; CRA, colorectal adenoma; AUC, area under the curve; gFOBT, guaiac fecal occult blood test; FIT, fecal immunochemical test for hemoglobin; CTC, CT colonography; EV, extracellular vesicle; mRNA, messenger RNA; lncRNA, long non-coding RNA; circRNA, circular RNA; exLR-seq, extracellular vesicle long RNA sequencing; d-signature, diagnostic signature; TEM, transmission electron microscopy; TPM, transcripts per million; DEG, differentially expressed gene; FC, fold change; mRMR, minimum redundancy maximum relevance; IFS, incremental feature selection; SVM, support vector machine; OS, overall survival; DFS, disease-free survival; ssGSEA, single sample gene set enrichment analysis; ANOVA, analysis of variance; NA, not available; PMBC, peripheral blood mononuclear cell; ROC, receiver operating characteristic; CEA, carcinoembryonic antigen; NKT cell, natural killer T-cell.
