# Supplementary material for: Plasma Extracellular Vesicle Long RNAs Have Potential as Biomarkers in Early Detection of Colorectal Cancer
Source: Front Oncol. 2022 Apr 8;12:829230. doi: 10.3389/fonc.2022.829230 (PMC9037372; doi:10.3389/fonc.2022.829230)
Supplement: Supplementary file 1 [file DataSheet_1.docx]

**Supplementary Materials**

**Plasma sample collection**

Blood samples of CRC and CRA patients were collected before the excision of tumor. About 8 ml peripheral venous blood was collected using EDTA-coated vacutainer tubes. Plasma was seperated within 2 hours after the blood collection. The peripheral blood was centrifuged at 3000 rpm for 10 minites at room temperature (15–25 °C), supernatant liquid was collected with 2.0 ml EP tubes and then centrifuged at 13000 rpm for 10 minites at 4°C. Supernatant plasma samples were collected and stored at –80 °C.

**EVs purification**

The exoRNeasy Serum/Plasma Kit (Qiagen, Hilden, Germany) was used to purify EVs following the manufacturer’s instructions. The thawed plasma was mixed with binding buffer and added to the exoEasy membrane affinity spin column then. For transmission electron microscopy, size distribution measurement and Western blotting, the EVs were eluted with elution buffer and then ultrafiltrated to concentrate. For the EV RNA isolation, we used QIAzol (Qiagen) to lyse the EVs and the total RNA was eluted and purified.

**Transmission electron microscopy (TEM)**

The purified exosomes were diluted with PBS and then concentrated by ultrafiltration (Amicon Ultra-15 tubes, Millipore). The concentrated EVs were fixed with 4% paraformaldehyde and a total of 10 μL of the resuspended EVs were transferred to a copper mesh for 3 minutes. The fluid was then absorbed from the edges of the mesh with filter paper. Then the mesh was stained with 1% uranyl acetate for 1 minute. After the staining solution was absorbed with filter paper, the sample was further dried for 2 minutes under incandescent light. The copper mesh was then photographed using a transmission electron microscope (JEM-1200EX, JEOL Ltd, Japan).

**Size distribution measurement**

The size distribution was analyzed using Flow NanoAnalyzer (NanoFCM Inc., Xiamen, China). The isolated EVs were diluted 1 to 100 with distilled water. We used the Silica Nanospheres Cocktail (S16M-Exo, NanoFCM Inc.) as the size standard to construct a calibration curve regarding particle sizes and side scattering intensities. The side scattering intensity of each vesicle was converted into the corresponding vesicle size.

**Western blotting of EVs**

We used Lymphoprep (STEMCELL Technologies, USA) to isolate peripheral blood mononuclear cells (PBMC). PBMC and EVs were lysed in RIPA buffer (1% NP40, 0.5% deoxycholate, 0.1% sodium dodecyl sulfate [SDS] in Tris-buffered saline) with complete protease inhibitors on ice for 30 min. Equal amounts of protein from EVs and PBMC were separated on 10% SDS-polyacrylamide gels and then transferred to nitrocellulose membranes (Bio-Rad, Hercules, CA, USA). Membranes were blocked with 5% non-fat milk and incubated with anti-CD63 (ab92726, 1: 1,000) (Abcam, Cambridge, USA), anti-TSG101 (sc-13611, 1:500) (Santa Cruz Biotechnology, Dallas, TX, USA), and anti-Calnexin (10427-2-AP, 1: 1,000) (Proteintech, Rosemont, IL, USA) overnight. Target proteins were detected using an enhanced chemiluminescence kit (Amersham Pharmacia Biotech, Uppsala, Sweden).

**RNA isolation**

We used miRNeasy (RNeasy MinElute spin column) included in exoRNeasy Serum/Plasma Kit to isolate total EV RNAs following the manufacturer’s instructions. The isolated EVs were lysed on the column using QIAzol (Qiagen), and total RNA was then eluted and purified. The isolated EV RNAs were subjected to RNA-seq library preparation or stored at -80 °C.

**RNA-seq library preparation**

DNase I (NEB) was used to remove DNA in the EV RNAs. Strand-specific RNA-seq libraries were prepared using the SMARTer® Stranded Total RNA-Seq Kit - Pico Input Mammalian (Clontech, USA). Then the cDNA was pre-amplified and R-probes with ZapR was used to deplete the ribosomal and mitochondrial cDNA. Next, purified dsDNA was subjected to 13-16 cycles of PCR amplification. Quality control of the libraries was conducted using Qubit (Thermo Fisher Scientific, USA) and Qsep100 (BiOptic Inc., Hangzhou, China). Further, the libraries were sequenced by the Illumina sequencing platform on a 150 bp paired-end run.
